# Integrative systems medicine approaches to identify molecular targets in lymphoid malignancies

**DOI:** 10.1186/s12967-016-1018-2

**Published:** 2016-09-01

**Authors:** Raffaele Frazzi, Charles Auffray, Angela Ferrari, Perla Filippini, Sergio Rutella, Alfredo Cesario

**Affiliations:** 1 Laboratory of Translational Research, IRCCS “Arcispedale S. Maria Nuova”, Reggio Emilia, Italy; 2 European Institute for Systems Biology and Medicine (EISBM), Paris, France; 3 Division of Hematology, IRCCS “Arcispedale S. Maria Nuova”, Reggio Emilia, Italy; 4 Division of Translational Medicine, Sidra Medical and Research Centre, Doha, Qatar; 5 John van Geest Cancer Research Centre, College of Science and Technology, Nottingham Trent University, Clifton Campus, Nottingham, NG11 8NS UK; 6 Clinical Governance and International Research Activities, Fondazione Policlinico Gemelli, Rome, Italy; 7 Division of Thoracic Surgery, Università Cattolica del Sacro Cuore, Rome, Italy; 8 European Association of Systems Medicine, Aachen, Germany

**Keywords:** Systems medicine, Lymphoproliferative disorder, Massively-Parallel sequencing, High throughput RNA sequencing

## Abstract

Although survival rates for lymphoproliferative disorders are steadily increasing both in the US and in Europe, there is need for optimizing front-line therapies and developing more effective salvage strategies. Recent advances in molecular genetics have highlighted the biological diversity of lymphoproliferative disorders. In particular, integrative approaches including whole genome sequencing, whole exome sequencing, and transcriptome or RNA sequencing have been instrumental to the identification of molecular targets for treatment. Herein, we will discuss how genomic, epigenomic and proteomic approaches in lymphoproliferative disorders have supported the discovery of molecular lesions and their therapeutic targeting in the clinic.

## Background

The growing medical needs in terms of cost reduction, care of elderly patients, increasing quality requirements and management, pose new hurdles to health professionals. Systems medicine (SM) is a fascinating approach to provide answers to these challenges. SM can be defined as an interdisciplinary, pro-active approach to the treatment of diseases that follows the P4 paradigm: predictive, preventive, personalized and participatory [1, 2]. SM takes advantage of a systems biology approach, whereby biological, clinical, environmental and lifestyle information is integrated through iterative statistical analyses, computational modelling and experimental validation [1].

A variety of hematological malignancies have been characterised, at least partially, using whole genome sequencing (WGS), whole exome sequencing (WES), transcriptome or RNA sequencing, including acute and chronic leukemias and lymphomas. The data derived from these efforts have provided pathogenetic insights, as well as diagnostic and therapeutic information, and they have identified molecular lesions amenable to targeted interventions. Next generation sequencing (NGS) or massively parallel sequencing refers to a technology which does not depend on traditional electrophoresis-based Sanger sequencing (i.e., the first generation) [3]. NGS platforms can be broadly divided into second generation (i.e., methods requiring template amplification before sequencing) and third generation (i.e., methods that sequence templates directly without amplification). Sophisticated computational tools are required to map and assemble the massive amount of data generated from a single NGS reaction.

The management of chronic lymphoproliferative disorders is a considerable burden on public health [4]. Non-Hodgkin lymphomas (NHL) are most frequently diagnosed in patients aged 65–74 (median age at diagnosis being 66 years) and account for 4.3 % of new cancer cases in the US [5]. NHLs are, therefore, the seventh most common type of cancer diagnosed in the US. The population age is increasing in Western countries, implying that the impact of healthcare costs necessary to cure these malignancies will be increasing as well. Death rates have been declining by 2.6 % on average each year during 2002–2011. The 5-year survival rate for NHLs currently approaches 70 % [5]. Specifically, survival rates for follicular lymphoma (FL) increased from 59 to 74 %, and those for chronic lymphocytic leukemia (CLL)/small lymphocytic lymphoma (SLL) from 66 to 69 % [4]. Similarly, EUROCARE and HAEMACARE data on 560,444 patients with haematological malignancies show an increased survival for most disease types in European countries, with the largest increase being for diffuse large B-cell lymphoma (DLBCL) and FL. However, persistent differences in survival across Europe point to the need for minimizing variations in the quality of care and availability of new treatments [4].

NHLs comprise over 60 disease subtypes and include both aggressive and indolent lymphoma subgroups. Approximately 95 % of lymphomas in the Western world are of B-cell origin. This overrepresentation largely originates from misrepair of DNA lesions introduced by activation-induced cytidine deaminase (AID), a B cell-specific cytidine deaminase that initiates class switch recombination and somatic hypermutation (SHM) of immunoglobulin (Ig) genes [6]. The three most common subtypes of NHL are represented by DLBCL, FL and CLL/SLL [7]. This article will discuss how genomic, epigenomic and proteomic approaches have supported the discovery of molecular targets in well-established lymphoid malignancies [8]. The main studies covered in this paper have been summarised in Table 1.

**Table 1:** Main studies using high-throughput approaches for the molecular classification and prognostic stratification of lymphoproliferative diseases

# of patients/disease type	Platform	Main finding(s)	Clinical implication(s)	Reference(s)
414 DLBCL	GEP (Affymetrix U133 plus 2.0 microarrays)	Stromal-1 and stromal-2 signatures	Stromal-1 associated with better outcome	[14]
203 DLBCL	Array CGH; GEP (Affymetrix U133 plus 2.0 microarrays)	Three molecular subtypes (GCB, ABC, PMBL)	Two MCRs restricted to ABC-DLBCLs predict adverse survival	[13]
127 DLBCL	Sequencing (Illumina GAIIx and HiSeq 2000)	109 genes with clear evidence of somatic mutation	Post-transcriptional modifications of histones act as core driver events in NHL	[16]
6 DLBCL (discovery); 73 biopsies (screening)	Massively parallel sequencing; WES; copy number analysis	Alterations of chromatin-modifying enzymes and genes involved in immune recognition by T cells	Drugs targeting pathways selectively disrupted in DLBCL subtypes	[17]
140 DLBCL	Genomewide DNA methylation profiling	Methylation disruption is a main epigenetic event	Extent of methylation variability is associated with survival outcomes	[19]
Various types of mature B-NHL, including FL, Burkitt lymphoma, marginal-zone lymphoma and CLL	Sequencing and mutation analysis	Inactivation of CREBBP/EP300 represents a common event in FL and DLBCL	Rationale for the use of histone deacetylase inhibitors in B-NHL	[18]
893 DLBCL	GEP; tissue microarrays	One-third of DLBCL demonstrate MYC/BCL2 coexpression	MYC/BCL2 coexpression more common in ABC-DLBCL and associated with aggressive clinical course	[24]
10 FL	WGS; WES	Early driver mutations in chromatin regulator genes (CREBBP, EZH2 and MLL2); mutations in EBF1 and regulators of NF-κB signaling (MYD88 and TNFAIP3) gained at transformation	Therapies targeting genetic alterations in a common progenitor clone are attractive	[44]
1274 CLL	Mutational and cytogenetic analysis	Four prognostic subgroups (high-risk, harboring *TP53* and/or *BIRC3* abnormalities; intermediate-risk, harboring *NOTCH1* and/or *SF3B1* mutations and/or del11q22-q23; low-risk, harboring 12 or a normal genetics; and very low-risk, harboring del13q14 only)	Differences in 10-year survival (29 % for group 1; 37 % for group 2; 57 % for group 3 and 69.3 % for group 4)	[48]

*GEP* gene expression profiling, *GCB* germinal center B-cell-like, *ABC* activated B-cell-like, *PMBL* primary mediastinal B-cell lymphoma, *MCR* minimal common region

### Use of high-throughput approaches to identify molecular targets in DLBCL

DLBCL is an aggressive type of NHL, accounting for approximately 30 % of adult NHLs diagnosed in Western countries [9]. A breakthrough in the treatment of DLBCL was represented by the use of the monoclonal antibody rituximab in addition to standard CHOP chemotherapy (R-CHOP), which led to shortening of time to disease progression and to dramatic improvements in overall survival (OS) [10, 11].

The cell of origin is an important prognosticator in DLBCLs. Gene expression profiling (GEP) has allowed the identification of three molecular subtypes of DLBCLs, i.e., germinal center B-cell-like DLBCL (GCB), activated B-cell-like (ABC) DLBCLs, and primary mediastinal B-cell lymphoma (PMBL) [12]. High-resolution, genome-wide copy number analysis coupled with GEP has indicated that GCB, ABC and PMBL are different diseases that use distinct oncogenic pathways and have a different prognosis [13].

GEP studies contributed further to the molecular and prognostic classification of DLBCLs through the identification of “stromal-1” and “stromal-2” signatures in a training group as well as a validation cohort of patients [14]. Molecular signatures were related to the microenvironment and predicted either a favorable outcome (stromal-1) by reflecting extracellular matrix deposition and macrophage infiltration of the tumor or a less favorable outcome (stromal-2) as a result of tumor blood vessel density. The stromal-1 and stromal-2 signature genes were more highly expressed in the nonmalignant CD19^−^ fraction compared with CD19^+^ lymphoma cells. These signatures were found both in GCB-DLBCL and in ABC-DLBCL, reflecting biological attributes of both subtypes of DLBCL [15].

At genetic level, massive parallel sequencing techniques revealed a remarkable complexity of DLBCLs represented by translocations, gene amplifications, single nucleotide variants but also copy number variations. Interestingly, recent genomic studies unveiled lesions affecting histone/chromatin modification enzymes, such as *CREBBP*, *EP300*, *MLL* and *EZH2* [16–18]. A further layer of complexity is represented by methylation profiles analyzed at the genome level. Methylation variability profiles (MVP) and methylation variability scores (MVS) have been recently introduced as novel methods to measure methylation disruption in lymphomas [19]. These parameters describe the deviation of the methylation patterns of DLBCLs from normal GCB cells (NGCB). They have been recently introduced and define six separate clusters in a cohort of 140 DLBCL analyzed. The magnitude of the methylation deviation from NGCB associates with survival of patients receiving R-CHOP, insofar patients with a larger magnitude of methylation changes display poorer survival compared with patients with smaller magnitude of methylation changes [19].

*MYC* oncogene rearrangements impart an unfavorable prognosis on lymphomas [20, 21]. Furthermore, the prognostic impact of *MYC* dysregulation is strongly influenced by concomitant *BCL2* aberrations. The concurrent translocation of *MYC* and *BCL2* occurs in approximately 5 % of DLBCLs and defines “double-hit” lymphomas that are treatment-refractory, with a median survival of ~8 months [22, 23]. Dual expression of these proteins is more common in ABC-DLBCLs and may explain its poorer survival [24]. This evidence points to the importance of assessing DLBCL patients for concurrent deregulation of *MYC* and *BCL2* at time of diagnosis, both at the level of translocations and protein overexpression [15].

Our knowledge of the biological heterogeneity of DLBCLs has prompted the development of novel targeted therapies, that have the potential for greater tumor specificity and lower generalized toxicity. An example is represented by agents that target B-cell receptor (BCR) signaling in DLBCLs. For instance, members of the NF-κB pathway are constitutively activated in ABC-DLBCLs [25, 26]. The activation of NF-κB in ABC-DLBCLs consists of an amplification of upstream oncogenic signalling, since it creates multiple feed-forward and feed-back signaling loops through transcriptional activation of target genes required for survival [27]. ABC-DLBCLs with an activated JAK/STAT signaling have a higher NF-κB target gene expression [12]. Interference with NF-κB activity induces cell cycle arrest and triggers apoptosis. In this respect, small-molecule inhibitors blocking the degradation of the NF-κB inhibitor IκBα or the activation of IKK are reportedly toxic for ABC-DLBCLs [28, 29].

The transcription factor IRF4 is, among others, a key player insofar it supports the survival of ABC-DLBCLs and, together with BAFF, is required downstream of BCR signaling to promote transcriptional programs during germinal centre formation [30].

B lymphocytes express a surface membrane B-cell receptor (BCR), which comprises a canonical immunoglobulin (heavy and light chains) coupled to a heterodimer CD79a/CD79b (Igα/Igβ), which is required for plasma membrane expression, intracellular trafficking and signal transduction. A distinctive characteristic of the BCR is that ABC-DLBCLs often express IgM-BCR on their surface, whereas GCB-DLBCLs typically show IgG-BCR switching [31]. There are inactivating events that specifically occur in the productive (in-frame) IgH allele in ABC-DLBCLs, whereas the non-productive (out of frame) IgH alleles switch to a different isotype [32]. This leads to a selective pressure on ABC-DLBCLs to maintain an IgM isotype in the IgM-BCR. The IgM- and IgG-BCR induce qualitatively different signaling cascades and, ultimately, transcriptional programs.

The CARD11 cytoplasmic scaffolding protein is a *bona fide* oncogenic protein that, when mutated, contributes to constitutive activation of NF-κB and enhanced NF-κB activity upon antigen-receptor stimulation [33]. The CARD11 protein is part of the so-called “CBM-complex” that is critical to the activation of the classical NF-κB pathway downstream of B- and T-cell antigen receptors [34]. The CBM complex mediates signaling from BCR to NF-κB. The Bruton tyrosine-kinase (Btk) is critical for the survival of ABC cell lines with wild-type CARD11, but not GCB lines or ABC lines with mutated CARD11 [35]. Ibrutinib is a novel inhibitor of Btk that has significant antitumor activity towards relapsed and refractory CLL, mantle-cell lymphoma and is currently in clinical trials for DLBCL [36, 37]. It is an irreversible inhibitor that forms a covalent bond with cysteine 481 adjacent to the active site of Btk, and has a high degree of specificity and potency. The better clinical response of ABC-DLBCLs to ibrutinib compared with GCB-DLBCLs supports the hypothesis that ABC-DLBCLs, but not GCB-DLBCLs, rely on the chronic activation of BCR signalling [38].

Another mechanism leading to the BCR-independent activation of the NF-κB pathway is mediated by the adapter MyD88, which is an inducer of NF-κB activity upon Toll-like receptor (TLR) stimulation of immune cells [39]. Mutant MYD88 isoforms, as well as ectopic expression of MyD88 mutants in heterologous cells, are potent activators of NF-κB [39].

Bortezomib is an NF-κB targeting drug that blocks the degradation of IκBα and has shown to benefit patients with relapsed ABC-DLBCLs, when combined with dose-adjusted EPOCH-R chemotherapy [40].

### Use of high-throughput approaches to identify molecular targets in FL

FLs have features of germinal center B cells and are currently considered incurable diseases when treated with conventional chemotherapy or immunotherapy. Understanding the evolution dynamics of FL clones is crucial to monitor disease progression and to effectively target FLs. Whole-genome and transcriptome sequencing of *BCL2* translocation-positive FLs in comparison with *IG*-*MYC* translocation-positive Burkitt lymphoma and normal germinal center B-cell samples showed differential methylation of intragenic regions that strongly correlated with expression of genes active in germinal center dark-zone and light-zone B cells [41]. These data point to a close connection between somatic mutation, DNA methylation and transcriptional control in B-cell pathways in FLs and other germinal center B-cell lymphomas.

Genetic profiling of serial FL biopsies suggests that FLs may derive by divergent evolution from a common ancestor cell. The pattern of somatic hypermutation of the *Ig* gene provides useful information about B-cell clonal evolution within the germinal center and also allows to discriminate B cells that entered the germinal center and display features of ancestor cells from B cells that continue to re-circulate across different lymphoid organs. The pattern of somatic hypermutation of the heavy chain of the immunoglobulin gene (*IgH*-*VH*) was examined using the GS-FLX Titanium sequencing platform in flow-sorted B cells of various differentiation stage, obtained from lymph node biopsies of FL patients with various patterns of evolution [42]. The authors reported a high level of clonality, with hundreds of distinct tumor subclones being detected in different cell subpopulations from the same patient sample. By using a lineage trees analysis, the oligoclonal FL population in all cases was shown to reside in an intermediate stage of maturation that maintains the capacity to undergo SHM, but is unable to further differentiate.

Intraclonal heterogeneity in FLs is supported by studies of SHM caused by AID in *IGH*. Aberrant SHM is defined as AID activity outside of the *IG* loci and mainly targets noncoding regions, inducing “passenger” mutations, but also having the potential to generate rare “driver” mutations. Ultradeep sequencing (>20,000-fold coverage) has been used to define the quantitative relationship between SHM and aberrant SHM on IGH (~1650 nt) and nine other noncoding regions potentially targeted by AID [43]. Single-nucleotide variants (SNVs) could be detected in 12/12 FL specimens. The aberrant SHM SNVs were associated with AID motifs. The relative number of SNVs with variable allele frequency <5 % varied with clinical grade, suggesting that tumor heterogeneity based on aberrant SHM may serve as a clinically relevant parameter.

Another study of WGS or WES followed by deep sequencing of 28 genes led to the identification of recurrent FL mutations in linker histone, JAK-STAT signaling, NF-κB signaling and B-cell developmental genes [44]. Longitudinal analyses allowed the detection of early driver mutations in chromatin regulator genes, whereas mutations in EBF1 and regulators of NF-κB signaling were gained at transformation. The remarkable intratumoral diversity within FLs is supported by studies showing a clonal hierarchy when comparing diagnosis and relapse tumor pairs, resolved using immunoglobulin somatic mutations and IGH-BCL2 translocations [45]. Using this approach, early versus late genetic events were clearly distinguished during lymphomagenesis. More specifically, IGH-BCL2 translocations and CREBBP mutations may be early events, whereas MLL2 and TNFRSF14 mutations may represent late events during FL evolution.

### Use of high-throughput approaches to identify molecular targets in CLL

CLL is a common B-cell malignancy characterized by a highly variable clinical course. A “watch-and-wait” therapeutic approach has long been advocated for patients without symptomatic disease, with frontline chemotherapy being a conventional choice for patients requiring treatment. More recently, new drugs have been approved for the treatment of patients with CLL, including novel CD20-targeting antibody obinutuzumab, PI3-kinase inhibitor idelalisib, and irreversible inhibitor of Bruton tyrosine kinase ibrutinib [46]. Our understanding of CLL genetics has been furthered by large-scale studies of massively parallel sequencing, which have unveiled the genetic and epigenetic heterogeneity among patients, and within individual patient samples. The most recurrent lesions identified, i.e., deletions of chromosome 13q (55 % of cases), 17p (7 %), and 11q (6–18 %); and trisomy 12 (12–16 %), reportedly contain putative CLL drivers, such as *ATM* and *BIRC3* in 11q, *TP53* in 17p, and miR-15a/16 encoded in an intron of *DLEU2* in 13q [46]. Other clinically relevant gene mutations in CLL involve *NOTCH1*, *SF3B1*, and *MYD88* [47]. It has been shown that addition of integrated mutational and cytogenetic information can improve prediction of OS in patients with CLL compared with cytogenetics alone [48].

The SHM status of the clonotypic rearranged immunoglobulin (Ig) heavy chain variable region (*IGHV*) gene is strongly associated with patient outcome [49]. Recently, the complete haplotype DNA sequence of the human Ig heavy-chain variable, diversity and joining genes was described. The authors also identified four large germline copy-number variants (CNVs) and eight CNV-containing haplotypes in addition to previously unmapped IGHV genes [50]. The international ImMunoGeneTics information system (IMGT^®^) has been enriched with these novel IGHV genes and alleles and the new sequences have been incorporated into a database and into reference directories [51]. The tool used to analyze the rearranged IGH sequences was the IMGT/highV-QUEST portal for NGS Ig and T-cell receptor sequences [52]. This is a high-throughput version of the IMGT/V-QUEST that meets the needs of deep-sequencing data analysis coming from NGS platforms, allowing for the simultaneous analysis of thousands of Ig and T-cell receptor sequences in a single run.

Genome-wide chromatin accessibility maps for CLL have been measured by the assay for transposase-accessible chromatin using sequencing (ATAC-seq) [53]. Significant differences in chromatin accessibility were shown for regions in the vicinity of genes that were identified as differentially expressed between *IGHV*-mutated and *IGHV*-unmutated CLL, allowing partial separation of two disease subtypes. *IGHV* mutation status could be predicted by the chromatin profiles and gene regulatory networks inferred for *IGHV*-mutated vs. *IGHV*-unmutated samples. This chromatic profiling assays should be straightforward to use in a clinical sequencing laboratory, allowing deeper insights into chromatic regulation in CLL as well as detection of biomarkers for stratified cancer therapy.

The presence of uracil into the DNA sequences was, until recently, considered the result of a misincorporation of dUMP during DNA replication and of spontaneous deamination of DNA cytosine. AID belongs to the family of APOBEC cytosine deaminases and represents a third source of uracil within the DNA sequence [54]. The DNA cytosine deamination by APOBEC-family enzymes is a natural event in both adaptive and innate immune responses through targeted deamination of Ig genes by AID and deamination of viral DNA by APOBEC enzymes, respectively [55]. Interestingly, recent data demonstrate that B-cell lymphoma cell lines contain several-fold increased levels of genomic uracil compared to normal human lymphocytes and non-lymphoma cell lines. Moreover, genomic uracil content correlated with AID protein expression and not with other APOBEC enzymes [54]. Analysis of exome sequencing data from lymphomas and CLL unravelled that these lymphoid malignancies may carry a distinct AID-hotspot mutational signature in kataegis regions, i.e., localized hypermutations in small regions that are also associated with genomic rearrangements [56]. Thus, AID-induced genomic uracil formation may be implicated in the development of localized hypermutation in B-cell malignancies [54].

A significant application of NGS allowed the assessment of the subclonal development based on mutations in the *IGHV*-*D*-*J* signature sequence in the dominant CLL clone [57]. This approach consistently evidenced, the occurrence of APOBEC and AID targeted mutations.

WGS studies in low-risk CLL patients with chromosome 13q deletion or normal cytogenetics showed that mutations in known CLL drivers are observed in only 33 % of this patient cohort, and are associated with normal cytogenetics and unmutated *IGHV* [58]. The *IGLL5* gene, which is homologous to IGLL1 (lambda5) and is critical for B-cell development, was the most commonly mutated gene and the mutational pattern suggested the potential involvement of AID activity. Three mutational signatures were identified, including two distinct AID processes (canonical AID and noncanonical-AID) that represent a greater fraction of mutational activity in mutated *IGHV* cases, and an ageing-related signature. The clonal or subclonal nature of a mutation was used to infer the time of occurrence of that mutation in relation to initial malignant transformation, the prediction being that clonal mutations occur earlier. Using this approach, noncanonical-AID-associated mutations were shown to occur in tumour evolution, whereas the ageing signature activity was enriched in patients with late-onset disease and enriched in subclonal mutations, which occur later [58].

The efficacy of WES approach in the discovery of driver mutations in CLL is further confirmed by the finding that 18 novel driver mutations were unveiled only with the use of this technique. These novel driver mutations hit histone proteins (*HIST1H1D*; *HIST1HIC*) involved in resistance to lenalidomide in multiple myeloma (*IKZF3*), nuclear RNA export factor 1 (*NXF1*) and proteins also mutated in acute myeloid leukemia and multiple myeloma (*ASXL1*; *TRAF3*) [59]. RNA sequencing (RNAseq) approaches were next used to validate the novel driver mutations emerged from WES analysis.

Proteomic analysis also represents a useful approach to the molecular classification of CLL. The mutational status of the *IGHV* genes defines two clinically distinct disease forms, known as mutated CLL (M-CLL) and unmutated CLL (UM-CLL). Patients with M-CLL usually have a favorable outcome, whereas patients with UM-CLL develop progressive disease and have shorter survival [60]. Quantitative proteomic analysis through the use of the iTRAQ-MS platform allowed the identification of two subsets of CLL clearly reflecting the IGHV mutational status. Specifically, 274 proteins were found to be differentially expressed when comparing UM-CLL and M-CLL samples. Proteomic analysis also allowed the establishment of a novel correlation between lymphadenopathy and UM-CLL, leading to the hypothesis that retention of malignant B cells in lymph nodes of UM-CLLs may underpin the development of lymphadenopathy [61].

Double *IGHV* gene rearrangements have been shown to correlate with patient prognosis in CLL [62]. Although differences in survival were not detected between patients with single or double *IGHV* chain rearrangements, the presence of at least one mutated *IGHV* gene conferred a better prognosis to patients with double *IGVH* rearrangements, suggesting that patients with double *IGHV* rearrangements with at least one mutated *IGHV* should be offered the same treatment intensity as those with a mutated rearrangement [62].

CLL is sensitive to immune system attack and is amenable to immune interventions, including the adoptive transfer of genetically modified T cells [63]. Rajasagi and co-workers [64] used WES and WGS to identify neoantigens presented by patient-specific HLA alleles. Neoantigens are generated from peptides encoded by gene alterations that are exclusively present in the tumor but not in normal tissues, and qualify as attractive targets for anti-tumor vaccination strategies. Using samples from 91 patients with CLL, 22 mutated binding peptides were predicted. In addition, HLA binding was experimentally confirmed for 55 % of such peptides. Neoantigen-specific T-cell responses were also monitored in CLL patients in continuous complete remission for >4 years after reduced-intensity hematopoietic stem cell transplantation (HSCT). In the first patient, 25 missense mutations were identified by WES. Patient T cells were expanded using antigen-presenting cells and peptide pools from the candidate neoantigen. Based on IFN-γ release, reactivity against the mutated peptides, but not against irrelevant peptides, was readily identified. In the second patient, 26 missense mutations were discovered and polyfunctional, highly avid memory T-cell responses were measured in vitro. Using mutant-specific tetramers, neoantigen-specific T cells could be detected and tracked in vivo coinciding with the achievement of molecular remission after HSCT.

Chimeric antigen receptor (CAR)-modified T cells have recently generated enthusiasm as an immunotherapy approach for lymphoproliferative malignancies [65]. Whereas CAR T cells typically target highly expressed antigens, low antigen-expressing tumours may develop resistance to CAR T-cell therapy. Interestingly, epigenetic modulators were shown to increase the expression levels of tumour-associated antigens, thus rendering tumour targets highly susceptible to immune system attack. The hypomethylating agent 5-aza-2′-deoxycytidine (DAC) induces NY-ESO-1, a cancer-testis antigen, in multiple myeloma and breast cancer cells in a time- and dose-dependent manner, with maximal effects being shown after incubation with 10 μM DAC for 72 h [66]. Single NY-ESO-1 peptides presented in the context of HLA-A*0201 molecules were increased by 100 and 50 % on MCF7 and U266 cells, respectively. This was reflected into an increased lysis of tumour targets by HLA-A*0201/NY-ESO-1_157–165_ peptide-specific CAR CD8^+^ T cells [66]. A similar approach has been used to increase the intensity of other tumour-associated antigens, i.e., mucin-1 (MUC-1) and prostate stem cell antigen (PSCA) [67]. In mice, complete eradication of pancreatic tumours is not achieved irrespective of the simultaneous challenge of pancreatic tumour cells (CAPAN1) with CAR T cells targeting both MUC-1 and PSCA [67]. However, CAPAN1 cells expressing low levels of MUC-1 as a result of immune escape mechanisms can be re-sensitised to the killing effect of CAR T cells after 4-day treatment with decitabine. Overall, these studies suggest that hypoethylating agents could be of benefit in the context of CAR T-cell immunotherapy trials that target a variety of tumour types.

### Mutational burden and immune checkpoint inhibitors in lymphoid malignancies

The use of immune checkpoint inhibitors for the treatment of haematological malignancies has raised considerable interest [68]. These agents do not target the cancer cells directly but rather enhance the cytotoxic activity of host T cells by blocking inhibitory signals from tumour cells. Cancer types harbouring more somatic mutations, i.e., lung cancer and melanoma, are expected to more frequently express neo-antigens that are recognized by autologous T cells, and should be more susceptible to checkpoint inhibitors [69]. It is presently unknown whether lymphoid malignancies may be less amenable to treatment with checkpoint inhibitors than solid tumours, based on the lower number of somatic mutations per megabase of coding DNA. Differences in response have been demonstrated across various haematological malignancies [70]. For instance, checkpoint inhibition is undoubtedly a promising strategy in classical Hodgkin lymphoma (HL). Recent analyses integrating high-resolution copy-number data and transcriptional profiles identified PD-L1 and PD-L2 as key targets of 9p24.1 amplification, which is a recurrent genetic abnormality in HL and may lead to overexpression of the PD-1 ligands on the Reed-Sternberg cell surface [71]. A recent study enrolled 23 patients with multiply relapsed or refractory HL, who received nivolumab (at a dose of 3 mg per kilogram of body weight) every 2 weeks until they had a complete response, tumor progression, or excessive toxic effects [72]. An objective response was reported in 87 % of patients, including 17 % with a complete response and 70 % with a partial response. The remaining 13 % of patients had stable disease. Analyses of pretreatment tumor specimens from a subgroup of ten patients showed copy number gains in PD-L1 and PD-L2 as well as increased expression of these ligands [72].

Immune checkpoint blockade has been shown to exert therapeutic activity in at least FL and DLBCL. A phase 2 MD Anderson Cancer Centre trial to investigate the activity of pidilizumab, a humanised anti-PD1 monoclonal antibody, with rituximab in 32 adult patients with relapsed FL showed objective responses in 66 % of evaluable patients, including complete responses in 52 % and partial responses in 14 % of patients [73].

A phase Ib study in relapsed or refractory B-cell lymphoma, T-cell lymphoma, and multiple myeloma has shown the efficacy of the anti-PD-1 monoclonal antibody nivolumab, with 40, 36, 15 and 40 % objective response rates among patients with FL, DLBCL, mycosis fungoides, and peripheral T-cell lymphoma, respectively [74]. By contrast, tumour regression responses were not observed among patients with multiple myeloma, with the exception of one complete remission occurring after local radiation therapy. The reason for the lack of objective responses in MM, compared with other tumors, remain speculative and may be related to immunosuppressive circuits within the myeloma tumour microenvironment [75]. Also, differences in the composition and quality of the immune infiltrate may account for differences in clinical responses across the wide spectrum of lymphoid malignancies.

### The emerging role of epigenomics

**Fig. 1:**
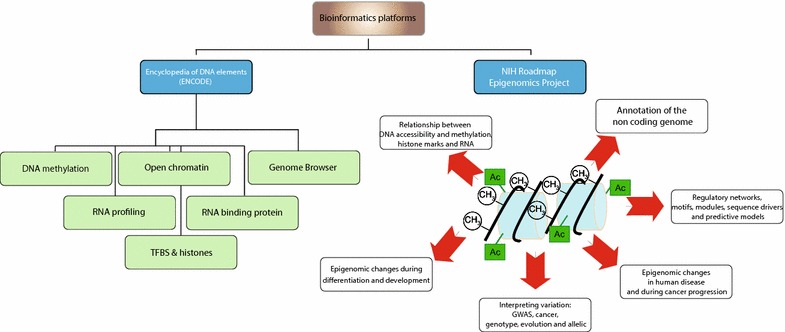
Molecular techniques and bioinformatics platforms for the epigenomic profiling of patients with cancer. TFBS is a set of integrated, object-oriented Perl modules for transcription factor binding site detection and analysis [87]. *GWAS* genome-wide association studies

Epigenetics and epigenomics focus on the study of chromatin, i.e., the complex of DNA, proteins, and non-coding RNAs (ncRNAs) that form the structural matrix of a chromosome. The spectrum of epigenetic modifications that regulate chromatin encompasses DNA methylation, histone modifications, and ncRNAs [76]. Epigenetic changes are modifications that do not alter the genomic nucleotide sequence but influence gene regulation and expression [77]. Changes involving gene expression control through epigenetic modifications are increasingly recognized as major mechanisms involved in the development of a variety of diseases, including cancer. These modifications can be reverted through pharmacologic treatment (such as small molecules, antibodies and inhibitors) and, therefore, identification of groups of patients with specific epigenomic alterations may lead to therapeutic benefits [78]. Furthermore, seven among the top ten leading causes of death listed by the World Health Organization in 2010 were diseases with an epigenetic and epigenomic component, underlying the need for boosting research efforts in this field [79]. The most updated molecular techniques and related bioinformatics platforms used for the epigenomic profiling of patients are shown schematically in Fig. 1.

DNA methylation is a key epigenetic mechanism used by higher eukaryotes for gene expression regulation, imprinting and silencing of germline specific genes and repetitive elements [80]. Methylation patterns are stably maintained through somatic cell division and can be inherited across generations. Since perturbed methylation patterns are linked to pathologic conditions such as imprinting disorders and cancer, understanding how these patterns are established and maintained is of great importance [81].

It is increasingly recognized that the epigenomic profiling of patients represents a useful tool in the path towards personalized medicine [77]. A growing body of evidence suggests that different molecular approaches should be integrated in order to build a realistic profile of the disease status of patients. Among these advanced technologies, epigenomic profiling is becoming a fundamental tool to explore gene expression regulation. Dysfunctional methylation patterns, for instance, may become the signature of a specific cancer whereas overexpression of specific deacetylases can be univocally associated with distinct malignancies. As epigenetic therapies advance towards the clinic, genomic/epigenomic tools will support the identification of patients who will most likely benefit from hypomethylating drugs.

Very informative in this context is the National Institutes of Health (NIH) Epigenomics Roadmap, a bioinformatics platform prompted by the NIH Roadmap Epigenomics Mapping Consortium [82]. This consortium developed a panel of resources based on NGS technologies in order to map DNA methylation, histone modifications, chromatin accessibility and small RNA transcripts in stem cells and primary ex vivo tissues that represent normal counterparts of tissues frequently involved in human disease. The NIH Epigenomics roadmap provides not only updated databases with the epigenomes of human cell types, tissues or whole organs but also protocols and reference guidelines for the scientific community.

Aberrant DNA methylation pattern is considered a hallmark of cancer, and may contribute to lymphoma phenotypes and chemoresistance of cancer cells. Decitabine is a nucleoside analogue that, after being incorporated into DNA, inhibits DNA methyltransferases (DNMTs) and acts as an hypomethylating agent. Decitabine has been approved for the treatment of myelodysplastic syndromes. A recent phase I study aimed at targeting aberrant DNA methylation in relapsed/refractory CLL and NHL by administering 1–3 cycles of decitabine [83]. Dose-limiting toxicity, consisting of myelosuppression and infectious complications, was documented in 2 of 4 patients with CLL and in 2 of 2 patients with NHL given decitabine at 15 mg/m^2^. Six patients received 10 mg/m^2^ decitabine for 10 days without DLTs and with no change in global DNA methylation or re-expression of methylated genes. When a 5-day decitabine schedule was evaluated, DLTs were observed in 2 of 6 CLL and 2 of 2 NHL patients, suggesting that dose escalation to levels associated with methylation changes may be difficult to achieve in patients.

The relevance of targeting DNMTs has also been explored in DLBCLs where chemoresistance is associated with aberrant DNA methylation programming [84]. Prolonged exposure to low-dose DNMT inhibitors reprogrammed chemoresistant cells and restored sensitivity to doxorubicin. A group of nine genes was found to be recurrently hypermethylated in chemoresistant DLBCL with SMAD1, a TGF-β pathway transducer, being an important contributor. A subsequent phase I study evaluated the impact of azacitidine priming for 5 days, followed by R-CHOP chemo-immunotherapy, in 12 patients with newly diagnosed DLBCL. Eleven of 12 patients achieved a complete response and ten remained in remission at median follow-up of 13 months. Patient biopsies obtained before and after treatment confirmed an increase of SMAD1 mRNA and a decrease in SMAD1 methylation, as well as a remarkable increase in the fraction of dead cells in the post-azacitidine samples. This study suggests that DNMT inhibitors may play a role in priming high-risk DLBCLs before administration of chemotherapy.

Decitabine has also been used in combination with vorinostat, a small molecule that binds to and directly inhibits histone deacetylase, and is approved for the treatment of cutaneous T-cell lymphoma. This phase I study enrolled 43 patients with advanced solid tumors and NHL [85]. Both sequential and concurrent schedules were evaluated in nine different dose levels (6 sequential and 3 concurrent). The sequential schedule of decitabine 10 mg/m^2^/day on days 1–5 and vorinostat 200 mg twice a day on days 6–12 qualified as the recommended phase II dose in this study. Disease stabilization for four cycles or more was observed in approximately 30 % of evaluable patients, suggesting that the combination of decitabine with vorinostat is tolerable and shows activity in different tumor types.

### Therapeutic implications of genetic findings

Recent insights into the molecular complexity of lymphoproliferative disorders are expected to herald breakthroughs in disease classification, prognostication and treatment. In addition to the major molecular designations of GCB and ABC subtypes, NGS technologies have unravelled the remarkable heterogeneity and complexity of DLBCL and identified molecular targets that may be selectively exploited for therapeutic benefit. Classifying NHLs based on cell-of-origin subtypes or presence of other molecular features portends important biological, prognostic and therapeutic implications. Agents targeting the oncogenic drivers of DLBCL subsets might expand the number of DLBCL patients achieving a cure, and are in various stages of investigation, as reviewed elsewhere [15, 28].

Molecular similarities between DLBCL and FL have provided insight into the potential of both NHL subtypes to respond to agents targeting common genetic alterations [86]. Despite differences in clinical behaviour, FL, similar to GCB-DLBCL, develops from GC B cells. Markers of prognostic significance may be relevant to both DLBCL and FL management [86].

Whole genome/exome sequencing has also been instrumental in disclosing the genetic landscape of CLL, revealing mutations of cancer-related genes and events involved in disease initiation and progression. BCR signalling is an attractive molecular target in patients with CLL, with the BTK inhibitor ibrutinib showing clinical activity [36, 47].

## Concluding remarks

The landscape of genomic and epigenomic changes associated with tumor transformation is increasingly disclosed due to the availability of new sequencing technologies and bioinformatic tools. Our appreciation of the heterogeneity of lymphoid tumors has grown following the use of high-throughput approaches. The unprecedented possibilities offered by these technologies will allow researchers and clinicians to develop novel and more effective treatment approaches. As the path towards integrative medicine and personalized treatments progresses, knowledge sharing through informatics databases will accelerate the dissemination and validation of experimental findings.

## Abbreviations

DLBCL: diffuse large B cell lymphoma
FL: follicular lymphoma
CLL: chronic lymphocytic leukemia
SLL: small lymphocytic lymphoma
SM: systems medicine
WGS: whole genome sequencing
WES: whole exome sequencing
NGS: next generation sequencing
GEP: gene expression profiling
DNMT: DNA methyltransferase
HSCT: hematopoietic stem cell transplantation
CHOP: cyclophosphamide, doxorubicin, vincristine, and prednisone
